# Assessing Socioeconomic Risks of Climate Change on Tenant Farmers in Pakistan

**DOI:** 10.3389/fpsyg.2022.870555

**Published:** 2022-05-31

**Authors:** Muhammad Tariq Yousafzai, Tariq Shah, Salim Khan, Sana Ullah, Muhammad Nawaz, Heesup Han, Antonio Ariza-Montes, Horacio Molina-Sánchez, Alejandro Vega-Muñoz

**Affiliations:** ^1^Centre for Management and Commerce (CMC), University of Swat, Swat, Pakistan; ^2^Department of Economics and Development Studies, University of Swat, Swat, Pakistan; ^3^Riphah School of Leadership, Faculty of Management Science (FMS), Riphah International University, Chakdara, Pakistan; ^4^Department of Environmental and Conservation Sciences, University of Swat, Swat, Pakistan; ^5^Institute of Business Studies, Kohat University of Science and Technology, Kohat, Pakistan; ^6^College of Hospitality and Tourism Management, Sejong University, Seoul, South Korea; ^7^Social Matters Research Group, Universidad Loyola Andalucía, Córdoba, Spain; ^8^Department of Financial Economics and Accounting, Universidad Loyola Andalucía, Córdoba, Spain; ^9^Public Policy Observatory, Universidad Autónoma de Chile, Santiago, Chile

**Keywords:** anthropogenic global warming, climate change, economic risks, tenant farmers, sustainable development

## Abstract

The study uses a transformative worldview to give voice to an economically marginalized group of tenant farmers vulnerable to climate changes due to their calamity prone geographical location. Drawing on anthropogenic global warming (AGW) theory lens, we examine the impact of manmade actions on climate change in District “Swat” and “Malakand” of Khyber Pakhtunkhwa (KPK) province, Pakistan using a sequential mixed methods research design. Through this research design, the results of quantitative survey were complemented with a qualitative analysis of in-depth interviews. In first phase, we conducted a survey of 200 tenant farmers, followed by second wave of data collection involving 12 open-ended in-depth interviews (IDIs). The both qualitative and quantitative results suggest that farmers in both districts are affected by climate change although their crop yield had progressively increased signaling better coping and survival skills than other parts of country. Majority of respondents believed that climate change is something beyond their control in disagreement with AGW theory. Major economic losses were specifically, due to sudden alterations in weather patterns, such as floods, and hailstorms that reduce productivity as well as results in food waste with no avenues available to reclaim the energy laden in organic food waste. Besides, a productivity loss was attributed to outdated farming, lack of awareness regarding sharecropping and crop loan insurance practices. The study concludes that farmers are most vulnerable to climate change in socioeconomic terms as such changes impact their income sources; This inwardly compels cash strapped tenant farmers to delve in practice of informal credit with substantive risks attached which further deteriorates their livelihoods. The study offers understanding of how low-literate and economically marginalized indigenous tenant farmers cope to climate change and offers policy recommendations to advocate for the rights to earn sustainable livelihoods in the face of grand climate challenge.

## Introduction

The developing countries are most vulnerable to climate change due to huge exposure and lack of awareness regarding impending threats (Ali et al., 2017; Rauf et al., 2018). Zhao et al. (2017) proclaims that global food security is disturbed due to altering rainfall spells and sudden rise in temperature in form of heat waves. Similarly, a study by Clapp (2017) asserts that food production at local level leads to food security indigenously. As a counter measure strategy to climate change, businesses now embrace the concept of Triple bottom line (TBL), which does not solely focus on profit dimension of performance. The TBL argues for a balanced approach to sustainable development by emphasizing social, economic, and environmental dimension of anthropogenic activities (Sun and Ertz, 2021). From a sustainable world perspective, some serious consideration is required to offset the effects of the grand challenge of climate change, which has the potential to act as an obstacle in attainment of sustainability (Sörqvist, 2016).

Due to direct interface of environmental conservation and agriculture, special attention is required to comply with quantifiable actions, policies, and programs as envisioned in sustainable development goals SDGs. On one hand, agriculture is a primal source of livelihoods of people in agri-based economies like Pakistan. On the other hand, this sector is most vulnerable to food insecurity threats due to climate changes (Dhimal et al., 2021). In Pakistan, a major stratum of the population, almost 42% is occupationally, related to agriculture that belongs mostly to rural localities (Shah et al., 2021). Pakistan is included in the list of top ten most vulnerable countries to climate change and was ranked among the three worst affected countries worldwide due to climate change (Kreft et al., 2013; Yousafzai et al., 2020). The reasons for such vulnerability are mainly attributed to arid and semi-arid climatic conditions and geographical dimensions. In comparison with humid regions, the impact of rise in temperature is more visible in arid and semi-arid regions (Huang et al., 2016). A rise in precipitation pattern has been witnessed ever since, the inception of new millennium, with an average value of rain recorded at 40% in South East, 20% in North, and 10% in central parts of Pakistan (Gitay et al., 2002). Since, the start of this century, Pakistan has confronted recurrent natural disasters. A destructive flood in 2012 adversely affected more than three million people including District Swat and Malakand in Khyber Pakhtunkhwa (KPK) province in Pakistan. As a result, thousands of hectares of agricultural land and more than four hundred lives were lost (Blunden and Arndt, 2012). Hence, in the backdrop of universal agreement on value of human life (Parncutt, 2019) the world needs studies on marginalized communities to mitigate their suffering by raising the voices of economically oppressed and environmentally threatened people. With this study, we aim to assess the socioeconomic risks of climate change on tenant farmers in Khyber Pakhtunkhwa and to assess their awareness levels to climate change from AGW theory lens.

It is common in academic discourse, that climate change affects farmer’s productivity on various dimensions, which inwardly cascades socioeconomic challenges as it affects their income. More specifically, such climatic variability affect the economically marginalized farmers more due to their limited skills sets and lack of adaptive capacities (Ofoegbu et al., 2017). The economic vulnerabilities of farmers in district Malakand and district Swat are important to study because such tenant farming practices differ from those of landholder farmers. Their informal contract arrangements are agreed prior to harvest seasons, which keeps farmers at a disadvantage in the absence of crop insurance schemes. Consequently, the tenant farmers are more vulnerable to climate changes due to the absence of credit support from Government and any other forms of support, such as crop insurance schemes Kabeer et al. (2010). Their socioeconomic conditions hinge upon a variety of factors beyond their locus of control that includes precipitation patterns, temperature, floods, and dry spells (Fahad et al., 2020). These climatic changes have become more challenging for the tenant farmers in both districts due to low awareness levels of tenants, which warrant the investigation of studies, such as this one to support the downtrodden tenant farmers in improving their climate resilience as significance of study. This will also help mitigate the socioeconomic shocks experienced by tenant farmers in target area including some districts of Malakand prone to tenant-landlord conflicts by informing policymakers to tailor effective programs interventions.

Assessing the socioeconomic impact of climate risks is important globally, due to its transboundary effects, which a challenge is for future generation of scientists. Parncutt (2019) contends that globally, climate change has become a serious threat to activities in the many sectors. Some of the neighboring countries in Asia, such as China, Nepal, and India, have successfully enhanced technical capabilities to counter climate change and thus reduced the negative effects of such changes on agricultural operations (Shabbir et al., 2019). These improvements are not without externalities due on bordering countries due to the transboundary movement emanating from burning of post-harvest residue in Indian to Lahore city of Pakistan. The situation was so alarming in certain areas of Lahore district due to smog that a lockdown was imposed as part of climate emergency response [Correspondent, 2021 (TNI, 2021 the news international)]. In this context, the government of Pakistan is also focusing on climate change and has made a Climate change (CC) policy to adhere the ideals of climate justice. Manifestations emanating from such a policy are evident from initiation of a “Billion Tree Tsunami Afforestation Project” which will also help improve local community needs in a sustainable manner (Kamal et al., 2019; Ullah et al., 2022). The Climate change policy of Pakistan aims to address a whole set of possible changes of adaptation and mitigation by serving as a focal document for related plans, projects, and programs for effective implementation. Unfortunately, Pakistan is one of the countries, which are strapped, in terms technical and financial support to deal with climate change. According to Global Climate risk index (2020), Pakistan is constantly ranked among top most vulnerable countries to catastrophes and adverse effects of climate change (Eckstein et al., 2019). Moreover, Khyber Pakhtunkhwa (KPK) province in Pakistan has suffered widely due to recurrent natural calamities, such as massive floods of 2010 and 2011, which swept away fertile soil and farmlands of locals (Ullah et al., 2019). It is clear that geographical location contributes to prevalence of such recurrent occurrence of catastrophes, such as heat waves, sudden rise in temperatures, and drastic changes in weather patterns in Pakistan (Yousafzai et al., 2020). In the backdrop of preceding decisions, it becomes imperative to search for better solutions to instill climate resilience and adaptation in local communities through improving the socioeconomic conditions of land less or land strapped tenant farmers. Hence, it is utmost necessary to adopt such countermeasure strategies to cope with this grand challenge and achieve SDGs oriented targets in true letter and spirit (Ahmed et al., 2020).

This study is conducted in district Malakand and District Swat, located in the north west of KPK province. Swat valley and adjoining Malakand districts are blessed with vast mountainous area with rivers flowing downstream lowlands of Khyber Pakhtunkhwa. This also makes both the districts prone to climate changes induced by floods (Saqib et al., 2018). Farming is one of the leading occupations for majority of population besides their own business, such as small enterprises like commodity shops; vegetable trading, fuel wood trading, and other trades associated with agriculture, such as seeds and fertilizer are common in study areas. A considerable portion of subsistence farmers have small land holding with tenant status and average land per tenant is estimated to be less than national averages. These people also lack access to formal sources of credit due to lack of collateral and livelihood assets (Saqib et al., 2018). Most common crops of the area are wheat, rice, sunflower, and maize. Different types of vegetable like potato, onion, tomato, okra and peas are also grown regularly. In fruits, peach, plum, apple, persimmon, and loquat are common.

**Figure 1:**
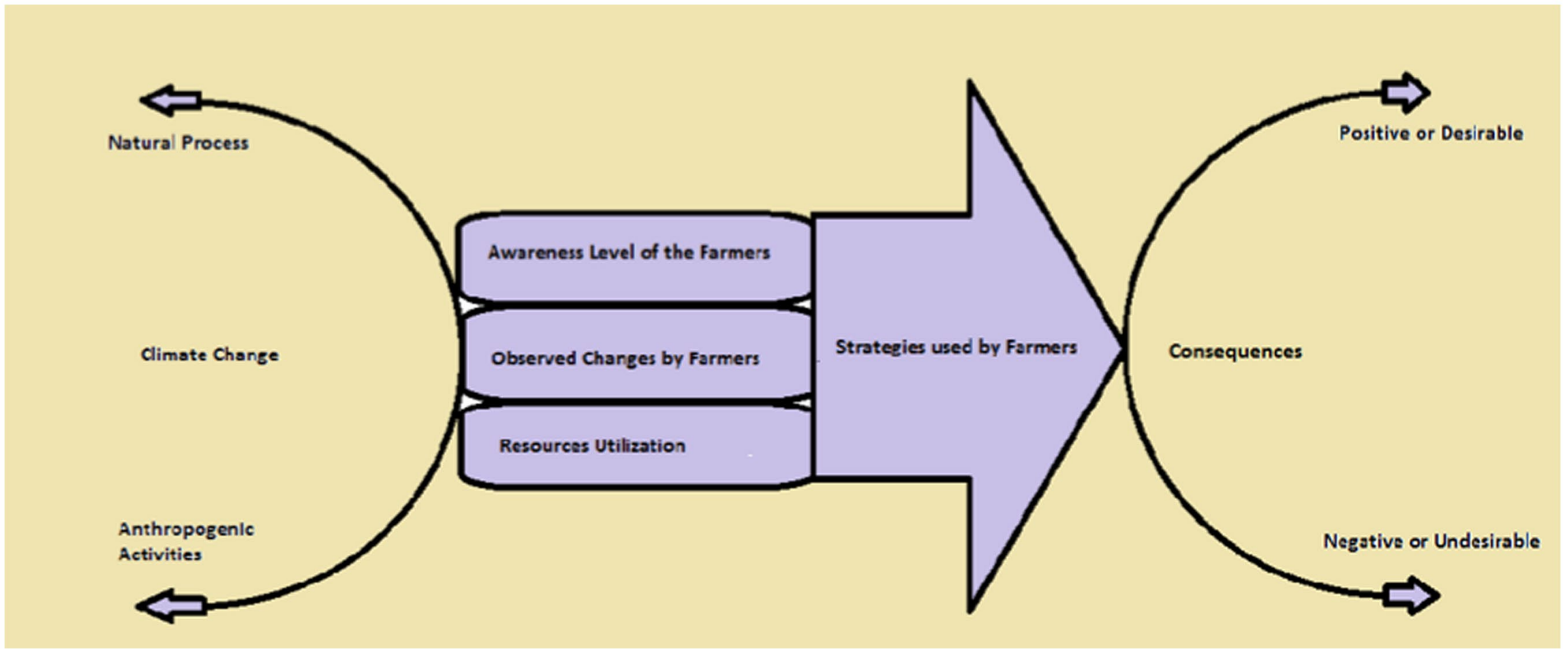
Conceptual framework of the study.

Majority of the studies conducted on the impact of climate on farmer’s livelihoods had used either a quantitative or qualitative research designs. This study is unique in a sense that it amalgamates both qualitative and quantitative approaches in a mixed methods research design in a rural setting of Pakistan as well as considers exclusively tenant farmers in land strapped regions of Swat Valley and protected areas of Malakand district in KPK, Pakistan. The study is important from a policy perspective as it informs the changes that had taken place in past two decades and contributes to original information available for future researchers in the areas of District Swat and Malakand in KPK. The aim of this research is to identify the overall impact of climate change on socioeconomics condition of the tenant farmers in Malakand and Swat district of Khyber Pakhtunkhwa province (KPK), Pakistan. The main objectives of this research are to:

- To assess the awareness level of the tenant farmers regarding climate changes.
- To determine concurrent changes due to climate change observed by such farmers in their areas.
- To investigates the adoptive strategies to mitigate the effect of climate change.
- Construct a policy guideline for policymakers and related stakeholders for overall improvement (Figure 1).

## Materials and Methods

According to Creswell and Creswell (2017) mixed methods are useful to understand complex problems, especially when there are contradictions between positivist (quantitative) and constructivist (qualitative) findings. We used a sequential mixed methods research design to achieve a more synergistic data collection and analysis across two stages of data collection to give voice to marginalized group of tenant farmers affected by climate change (Wisdom and Creswell, 2013). The study was conducted in two districts of Swat and Malakand in Khyber Pakhtunkhwa (KPK) which, were prone to natural calamities as evident from recurrent pattern of floods, hailstorms, strong winds, and dry spells. KPK is a topographically a diverse province in Pakistan. It has a versatile weather and a unique topography. Its climate varies from the dry and hot rocky zones in south to the cool and lavish green forests in the north. It has twenty-five (25) districts in total (Babar et al., 2015). For the study, a representative sample of 200 tenant farmers was selected through use of multistage sampling technique in both districts Swat and Malakand during initial survey phase followed up by twelve in-depth interviews during inductive second phase. In the former phase, a comprehensively structured questionnaire was used to collect information from farmers and in later phase, an interview protocol was used to collect information from participants. General demographic characteristics and data regarding the socioeconomic status of the respondents as well as details about climate change from farmers’ perspective. Special focus was made to analyze the changes in the cropping pattern due to climatic change.

The research onion was used to situate the research by depicting various layers encompassing worldviews, strategy of inquiry, and data collection approaches and procedures (Saunders, 2011). This study used a sequential mixed method research choice with a transformative worldview to raise voice for the economically disadvantaged tenant farmers who are involved in farming where nature of contracts is informal and verbally agreed prior several months in advance to harvest season. The nature of data collected across both quantitative and qualitative stages of sequential mixed methods can be termed longitudinal as it involves data collected over a span of two stages.

In the first phase, our research positionality was inclined toward a positivist worldview. In the second phase of inductive nature we used a social constructivist worldview with a qualitative analysis of 12 interviews from same set of respondents to offer a longitudinal stance of data collection although many other delimitations also favored collection of such information. In the first phase, we used Krejcie and Morgan methods table (Morgan, 1970) to determine adequacy of sample size. In the second phase, we used a purposive sampling procedure for recruitments of participants of study and used data saturation concept when new insights ceases to emerge from further data collection as indication of interview adequacy with 12 in-depth interviews (Creswell and Creswell, 2017). In order to maintain trustworthiness of data collected through interviews, we used member checks, peer debriefing, and follow-up interviews in qualitative part of study. In both phases of data collection, the investigator assured the participants regarding the privacy of their information. In the second phase of qualitative data as per the ideals of ethics, we used aliases to maintain privacy of participants (Iqbal et al., 2018).

**Figure 2:**
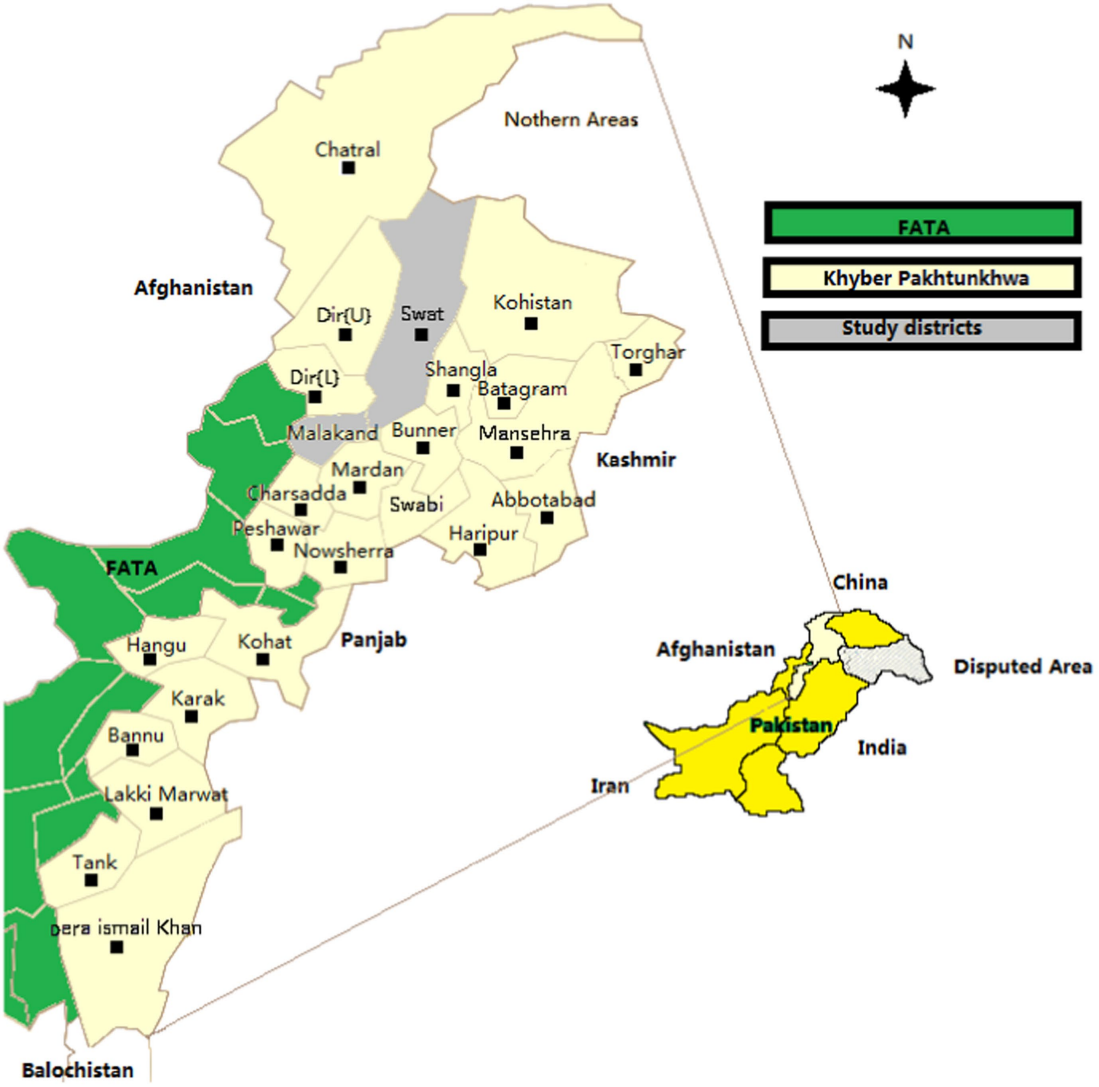
Study areas.

Pakistan’s province Khyber Pakhtunkhwa (KPK), formerly named North West Frontier Province (NWFP), is the area designated for this study as shown in Figure 2. Specifically, the two districts of District Swat and District Malakand as evident from Figure 2 used for this study. The KPK province has a versatile weather and a unique topography. Its climate varieties from the dry and hot rocky zones in south to the cool and lavish green forests in the north. It has twenty-five (25) districts in total in KPK (Babar et al., 2015). Maximum temperature of the northern region is also showing an increasing trend in case of Kharif as well as rabi seasons. The Kharif cropping season trend line shows a 0.7°C increase, whereas Rabi crop is showing a 2.5°C increase in Rabi season over the past 30 years. The overall increase in maximum temperature can be one of the factor causing increased rains, melting of snows, and glaciers that results in unalarmed floods in the lower part of KPK but the crop productivity trend in both the season is positive and favorable (Babar et al., 2015). Mean temperature trend of the northern region is almost stationary for the Kharif crop, whereas it is showing a sharply increasing trend in case of Rabi cropping season. An increase of 1°C is observed in the Rabi mean temperature. The increase in temperature is having a favorable impact on vegetation growth that has been positively affecting Rabi crops.

## Results and Discussion

This section will focus to illustrate the views and finding of the respondents in both the phases of mixed methods research study. In the first part, we discuss the results from survey followed by a qualitative analysis of in-depth interviews (IDIs). The context of the study is incorporated with relevant literature, which supports or opposes the finding of this study offering insights into how climate changes impacts socioeconomic conditions of tenant farmers in the study area. Farming is one of the prime occupations, which are generally affected by climate change (Rosenzweig et al., 2014). It is obvious from Table 1 that majority of the respondents in first phase of data collection were uneducated comprising almost 60% of the participants. Another study was conducted by Ali et al. (2017) which reveals 51% of the respondents as educated, while 49% were uneducated in their study in the northern areas of Malakand division comprising both Swat and Malakand Districts. A recurrent theme, which manifests during the thematic analysis of the data collected from IDIs, reflects that there is higher level of unemployment in Pakistan. Most people associated with farming are tenants who do not have their own land in study area. Hence, these economically marginalized people prefer farming or other informal apprenticeships instead of formal education. Moreover, their women also support farming activities in the confines of their four walls and their education level is even lower due to lack of financial support and access. However, as part of this study no women were interviewed due to cultural barriers of a conservative society.

**Table 1:** Impact of climate change on socioeconomic conditions of farmers.

**1. Status**	**District name**		**Total**	**Percentage**
	**Swat**	**Malakand**		
Educated	51	29	80	40
Uneducated	49	71	120	60
Total	100	100	200	100
**2. Knowledge regarding Change**	**District name**		**Total**	**Percentage**
	**Swat**	**Malakand**		
No	14	42	56	28
Yes	84	58	144	72
Total	100	100	200	100
**3. Long-term shifts in temperature**	**District name**		**Total**	**Percentage**
	**Swat**	**Malakand**		
No	18	0	18	9
Yes	182	100	182	91
Total	100	100	200	100
**4. Cool/warm**	**District name**		**Total**	**Percentage**
	**Swat**	**Malakand**		
Cool	16	1	17	8.5
Warm	84	99	183	91.5
Total	100	100	200	100
**5. Long-term shifts in precipitation**	**District name**		**Total**	**Percentage**
	**Swat**	**Malakand**		
No	10	0	10	5
Yes	90	100	190	95
Total	100	100	200	100
**6. Drier/wetter**	**District name**		**Total**	**Percentage**
	**Swat**	**Malakand**		
Drier	84	99	183	91.5
Wetter	16	1	17	8.5
Total	100	100	200	100
**7. Long-term shifts in drought**	**District name**		**Total**	**Percentage**
	**Swat**	**Malakand**		
No	10	2	12	6
Yes	90	98	188	94
Total	100	100	200	100

Most of people think that climate change is involuntary natural process beyond their locus of control. Fatalism and Passive resignation to events are common in farming communities of Muslim faith oriented countries like Pakistan (Mahmood et al., 2020). Some of the respondents also believe that climate change occurs with anthropogenic activities. Table 1 part 2 indicates information in response to knowledge of climate change, which reveals that majority of the respondents possess knowledge about climate change but this knowledge is not conceptually clear due to weak educational backgrounds. The finding by Maponya et al. (2013) suggested that about 63.3% people have received information regarding climate in South Africa. While, only 36.7% people did not receive any information in Mpumalanga Province, South Africa. These facts are also supported by the work of Maryam et al. (2014) who proclaim that in upper Swat region of Pakistan, people are well aware of their surroundings and have felt the changes in climate. They further argued that 94.8% of respondents observed changes in the climate and only a dismal 5.3% did not observed such changes. However, a recurrent theme, which manifests itself during qualitative thematic analysis reveals that majority of people are aware of the climate change but hitherto unable to comprehend the impact of such changes in totality. Due to their low-literacy levels, such people are skeptical of the Governmental narrative on climate change. They attribute the changes to a variety of factors beyond their control where human contribution is minimal in disagreement to anthropogenic global warming viewpoint.

The ensuing Table 1 part 3 express that majority of the respondents 91% observed a long-term shift in rise of the average temperatures in the study areas of District Malakand and Swat spanning more than 100 kilometers. In some regions, rising temperature would improve some crops, such as rice, for example, in Mediterranean areas, where cool weather usually leads low crop production (Nakagawa et al., 2003). But the negative effects associated with temperature increments heavily outweigh the positive ones (Shah et al., 2011). The findings of Moretti et al. (2010) indicate that extreme temperatures will impact fruit quality, such as fruit firmness, sugar, and antioxidant compounds. On similar lines, a recurrent pattern which emerges from qualitative thematic analysis of data collected from IDIs suggest that a rise in temperature-related events has been observed in both district Malakand and Swat which has adversely impacted the productivity of tenant farmers due to weather extremes. The spot rise in temperature is due to fact that combined area of both districts contains both upstream and downstream areas, which are far a part in terms of distance. Moreover, based on inputs from qualitative data, it is suggested that temperature rise has caused many people from other parts of country to migrate to upper areas of Swat and Malakand which had further reduced the land availability for farmers, which cascades negative vibes on their livelihoods due to limited land.

Information in Table 1 part 4 indicates that 91.5% of the respondents perceived warmer weather condition in their localities. Only a dismal 8.5% of the respondents, observed cooler weather condition in their areas mainly due to the vast distances between two districts as well as their upstream and downstream locations. Likewise, analysis of in-depth interviews (IDIs) reveals that the intensity of cold was high in 2020 and 2021 during the Corona pandemic mainly due to restriction in human activities. This is presumably attributed to reduction in emission levels due to lockdowns observed in past 2 years. Moreover, the qualitative interviews suggest that mountains have received greater level of snow in both District Swat and Malakand during the pandemic years (2020–2021). The general observation also supports this fact as the mountains in past 2 years had become greener due to excessive rain spells and snow received in the area. The analysis goes in agreement with notion of anthropogenic global warming, which entails that mainly human activities contribute to global warming and climate change (Fielding and Hornsey, 2016). However, there is a higher level of ambiguity relating to the fact that in the past cyclical rises and downturns were also experienced by the residents of the district Malakand and Swat.

There is a time in-variant relationship between precipitation and crop yield in the context of climate change (Feng et al., 2021). Among certain other consequences of climate change, precipitation pattern is crucial, which increases the probability of impacting farmers’ socioeconomic conditions. Erratic changes in precipitation pattern contribute to lower yields with a corresponding rise in temperatures or fall in temperatures (Bloomfield et al., 2006; Lobell and Burke, 2008; Robinson and Katherine, 2010). Information in Table 1 part 5 indicates that 95% of the respondents observed long-term shift in precipitation pattern in their localities. While, only 5% did not observe any changes in precipitation patterns mainly due to lower recall and recognition of such changes. Due to the shift in precipitation patterns as evident from Table 1, 91.5% of the respondents think that on aggregate weather condition is going drier. While, only 5.5% of them consider that weather condition on aggregate is moving toward wetter conditions with the passage of time in the target areas of study. This inwardly, shows that generally there is little water available for irrigation especially in downstream areas. However, a recurrent pattern, which emerges from qualitative analysis of in-depth interviews, entails that in the past three years (2019–2021), recursive rise has been experienced in precipitation amounts in district Malakand and Swat. A recurrent pattern from qualitative thematic analysis of in-depth interviews suggests that higher precipitation helps improve local crops but the accompanying hailstorms and strong winds generally mar farmers’ productivity of tomato, peach, and apples in district Swat and Malakand causes huge amount of food losses from orchard farms.

In the same vein, information presented in Table 2 part 1 shows the results of the measures of farmers for mitigating the impact of climate change. The information received in response to question revealed that 16.5% of respondents used alternate planting dates to reduce climate change impact. These results are in conformity with the findings of Lasco et al. (2014) who reported from a study conducted in Tanzania that farmer used planting date variation practice to adapt to challenges of climate change. Another finding reveals that 60% of the respondents used different crop varieties for adjustment of their fields to climate changes. The switching of crop varieties, that is, sowing one crop variety instead of other is the emerging response to climate change in the study areas. In a study by Komba and Muchapondwa (2012), it was reported that some farmers try to adapt climate change by planting drought-resistant crops in Tanzania. Similarly, using Avena (Indigo) species as a fodder crop which was lately replaced by a dominant stable crop (barley) performed as a means of adaptation to climate change (Menon et al., 2007). It was further reported by 8 and 15.5% farmers, respectively, that techniques, such as additional irrigation and crop rotation, are used as countermeasures for adjusting to climate change. To assure harvest in the face of climate change, farmers adapt cropping systems and management measures, for instance, by implementing different crop rotations, crop sowing dates, irrigation, and tillage methods (Kaukoranta and Hakala, 2008; Fleming and Vanclay, 2010; Daccache et al., 2012). Farmers are also likely to choose new, climatically suited crops or cultivations that are better adapted to warm and dry conditions, such as low delta crops (Bloomfield et al., 2006; Tokatlidis, 2013). Extreme weather events in the future will probably set yield at higher risks, which may lead to an increase in pesticide usage and fertilizer input (Baessler and Klotz, 2006; Lososová et al., 2006). On the contrary, the empirical evidence generated from in-depth interviews reveals that farmers use indigenous knowledge including the fog formation months, local calendars of farming months as well as the behavior of insects and animals to determine changes in weather triggered by climate change.

**Table 2:** Countermeasures to climate changes adopted by farmers.

**1. Adjustment of farmers to climate change**	**District name**		**Total**	**Percentage**
	**Swat**	**Malakand**		
Changing planting dates	10	23	33	16.5
Using different crop varieties	55	65	120	60
Adding irrigation	11	5	16	8
Crop rotation	24	7	31	15.5
Total	100	100	200	100
**2. Source of weather predict for next season**	**District name**		**Total**	**Percentage**
	**Swat**	**Malakand**		
Use past season weather	34	10	44	22
Expert opinion	36	61	97	48.5
TV/ radio	19	5	24	12
Internet	10	4	14	7
Others	1	20	21	11.5
Total	100	100	200	100
**3. Predict risk due to climate change**	**District name**		**Total**	**Percentage**
	**Swat**	**Malakand**		
Drought	62	61	123	61.5
Cyclone	2	3	5	2.5
Floods	12	24	36	18
Storms	9	2	10	5
Heavy rain	15	8	23	11.5
Others	0	2	2	1
Total	100	100	200	100
**4. Impact of climate change on**	**Increases (%)**	**Decreased (%)**	**No change (%)**	**Do not know (%)**
Crop Yield	61.5	29.5	8.5	0.5
crop quality	48	34	18	-
crop growth	36.5	51.5	11.5	0.5
crop disease	87.5	8.5	4	-
crop water requirement	89	9	2	-

Climate change is predicted to result in a higher frequency of extreme weather events, such as heavy storms, summer droughts, and extreme cold spells (Jentsch et al., 2009; Coumou and Rahmstorf, 2012). Table 2 section 2 indicates various sources that farmer use for predicting the future weather condition. It was found that 48.5% of the respondents relied on weather experts/meteorologists. Similarly, 22% of the farmers reported that they use their past experience. Out of the total sampled farmers, 12% of respondents used television and radio for weather prediction. Among the sampled farmers, only 7% reported the use of Internet for weather prediction. While 11.5% farmers reported that they use other sources for weather prediction in their area. Dessai and Hulme (2004) also reported that the costs of climate changes are differentiated by various types of risk. For instance, some risk is associated to the rate and magnitude of climate change, whereas some is associated to biological response of agricultural products and how the community responds. Climate change also influences weeds indirectly, by enforcing adaptations of farming methods, such as crop choice, sowing time, harvesting date, and other agronomical practices to these alterations (Fleming and Vanclay, 2010). The empirical evidence generated from interviews (IDIs) reveal a recurrent pattern whereof the farmers council (community level) and ongoing observation and discussions were used in coping up the climatic changes triggered by extreme weather events. Besides, use of Internet was more pronounced among young tenant farmers than others, although the overall theme reveals a mixed approach to information used for weather prediction.

Prediction of climate change risks for farmers’ livelihoods is crucial because it directly impacts farmers’ livelihoods. According to García-Martínez et al. (2016), almost 85% of small-scale farms are subsistence oriented and produces primarily for on-farm consumption, while only 15% farms are market-oriented with commercial objectives. Therefore, tenant farmers mainly seek family welfare (including education) and vulnerability reduction by applying low-cost coping strategies to climate change. It is expected that there will be decline in crop yield up to 30% by the mid of 21st century in certain regions (Rotz et al., 2016). As evident from Table 2 part 4 majority of (61.5%) respondents’ increased their crops yield, while 29.5 respondents witnessed a decline in yield of their crops due to climate change. This result goes in agreement with experienced a decline (White, 2012; Rotz et al., 2016). Our findings also showed that 48% of the farmers reported that there is an improvement in the crop quality due to climate change as the used of imported seeds has been in vogue recently. On the contrary, 34% of the farmers reported that crop quality has deteriorated due to changes in the climate. These results support the findings of Moretti et al. (2010) who reported that extreme temperatures will impact fruit quality, such as fruit firmness and sugar as well as antioxidant compounds. As evident from Table 2 part 4, 18% of the respondents did not observe any change on their crops quality. It was further observed that 36.5% of the tenant farmers disclosed that crop growth has been enhanced due to climate changes, especially due to the rise in rain spells. While, 51.5% of the farmers reported a decrease in the crop growths due to climate changes. In the same way, majority of respondents, that is, 87.5 and 89% reported increase in the crop disease and crop water requirement respectively, due to climate change while 8.5 and 9% of the respondents account decrease in the crop disease and crop water requirements, respectively, due to climate change. The empirical evidence generated from IDIs in qualitative data analysis complements the findings of preceding survey although the crop yield has been improved but not quality. The introduction of hybrid seeds has practically eradicated the natural taste crops and fruits as well reports of lower nutritional value have been observed in District Swat and Malakand.

Agricultural risks have been part of life since the beginning of the evolution of the human race. Valdés et al. (1986) indicated that risk and uncertainty pose a serious impediment to agriculture development. Such risks inwardly translate into socioeconomic risks as income of tenant farmers is disturbed due to them. Crop diversification, share cropping, and sharing food during times of scarcity are all part of risk management strategies. Information in Table 3 part 1 shows some of the risk related to farming in the study areas. A reasonable portion 42.5% of the farmers indicates that production risk is one of the leading risk in their regular farming which affects their income and hence their socioeconomic conditions. Similarly, a majority of 44.5% of the respondents depict price of their agricultural product is the prime risk in their areas as sudden fall and rise in market prices cascades economic losses and gains beyond their control. The prices of agricultural commodities are instable and subsequently changes from very low to very high in the study areas due to poor enforcement of laws and informal nature of market dynamics. Agricultural producers including tenant farmers have little or no control over the market forces that drive commodity prices. Information in the same table part 2 illustrates information of the respondents regarding the consequences or major losses due to climate changes. It is evident from the table data that majority of the respondents 45% express that drought or extended dry spells is one of the foremost losses to climate change in the areas especially for rainfed farms. On a global scale, this risk is much greater than that of cyclones, floods and storms experience in target areas of study. However, on a regional rather than global scale, there are areas where the risk of flooding exceeds that of drought. Similarly, losses of agricultural production reported by 34% of the respondents in the study areas. The theme which emerged from the qualitative analysis of in-depth interviews, confirms the preceding findings as tenant farmers experience adverse risks due to unfriendly weather changes due to which many of the orchard farmers experience bankruptcy. In socioeconomic terms unfortunately for tenant farmers, failing to fulfill obligations to land owners also results in social stigma. According to Lead Pakistan Under Play (2019) article the almost 25–30 percent of people in Malakand and Swat districts are by the use of Under Play to meet the demands of debtors. Under this scheme, the lender give items to borrowers at a higher cruel price while the borrower sells it on market price to extend financial life line up till next harvest season. The emergent themes from qualitative analysis confirm that majority of tenant farmers also delve in “underplay or Neta Watta” which refers to gamblers like risk taking in order to fulfill their unmet financial obligations to land lords and family needs.

**Table 3:** Information of the farmers regarding Agricultural risk and major losses due to climate change.

**Agricultural risk in study area**	**District name**		**Total**	**Percentage**
	**Swat**	**Malakand**		
Production risk	69	16	85	42.5
Price/market risk	29	60	89	44.5
Financial risk	2	23	25	12.5
Others	0	1	1	0.5
Total	100	100	200	100
**Major losses due to climate change in study area**	**District name**		**Total**	**Percentage**
	**Swat**	**Malakand**		
Heavy rainfall (floods)	15	11	26	13
Drought	51	39	90	45
Rising temperature	3	1	4	2
Loss of Agric. production	26	42	68	34
Others	5	7	12	6
Total	100	100	200	100

Information in Table 4 indicated the overall impact of climate change on some of the growing crops and orchards. It is evidence from the table that majority of the growing plants, crops, medicinal plant, and wild life including birds are adversely affected from climatic changes. Majority of the respondents confirm that the quantity of such plants, crops and wild life had decreased with the increase in climatic changes. Information in the given data further demonstrates strategies to mitigate the effect of climate change in their areas. Similarly majority of the respondents 72% and 75.5 highly desired that increase in the farmers knowledge and the provision of proper training for adoptive capacity, respectively, mitigate the effect of climate change in the study area. In the same vein, 66% of the farmers reveal that Advancement weather forecasting system will help to mitigate the effect of climate change. Moreover, 64% of the respondents highly desired that the provision agricultural finance would obviously mitigate the effect of climate change in the study area. Some of the respondents 29.5% desired that reduces the use of fertilizer will mitigate the impact of climate change.

**Table 4:** Impact of climate change on indigenous yield and mitigating strategies by the farmers.

**Impact of climate change on:**	**Increases (%)**	**Decreased (%)**	**No change (%)**	**Do not know (%)**
Medicinal Plants	5.5	88	5	1.5
Birds and wildlife	5	83	10.5	1.5
Pears trees	3.5	83	12.5	1
Apple trees	29	61	9	1
Wheat	38	53	9	0
Maize	15.5	75	9.5	0
Walnut trees	3	51	21	25
Honeybee colony	22.5	74	3.5	22.5
**Strategies to mitigate the effect of climate change**	**Not desired (%)**	**Less desired (%)**	**Somewhat desired (%)**	**Highly desired (%)**
Reduce the use of artificial fertilizer	27	11.5	32	29.5
Enhance the knowledge of farmers	1.5	2.5	24	72
Create fund research activity	2.5	9	31	56.5
Introduce crop insurance policy	15.5	6	19.5	59
Strengthen through agricultural finance	3	6	27	64
Advancement weather forecasting system	4.5	10.5	24	66
Provision of Training for adoptive capacity	4	4	16.5	75.5

Common views regarding climate change have their socioeconomic roots, which are specific to geographical locations, such as urban and rural localities (Weckroth and Ala-Mantila, 2022). The study has unveiled a variety of findings in rural areas of district Swat and Malakand. The study unveils that tenant farmers are affected in socioeconomic terms by the changes triggered due to climate change. In particular the under play mechanism used as a short-term fix causes severe issue for tenant farmers, especially in instances wherein they are unable to meet their agreed upon financial obligations to farmers. This has wide range of socioeconomic impact on farmers and their families. The study proposes better tenant farm owner contracts to safeguard the interests of economically marginalized tenant farmers in district Swat and Malakand. The farmers in target area although are disadvantaged due to lack of land provision but at the same time had witnessed a rise in production of yield although such rise in not proportionate to fulfill their socioeconomic needs. The farmers use a variety of indigenous countermeasures, such as crop managing by crop sowing dates, use of hybrid seeds but not without associated tradeoffs. It has been reported that hybrid seeds had altered the original taste of fruits and crops. A similar study was conducted by Maryam et al. (2014) in Swat District only by using a positivist’s world view to report that a whopping majority of people were aware of the perils of climate change on their livelihoods. Likewise, a study conducted by Maponya et al. (2013) in South Africa goes in agreement with our findings wherein 65% of people were aware of the dangers of climate change. Another study was conducted by Lasco et al. (2014) in Tanzania whose result goes in agreement with findings of this study in regard to socioeconomic impact of climate change of livelihoods. Likewise, Komba and Muchapondwa (2012) conducted a study to assess the climate adaptation of small farmers in Tanzania wherein farmers utilize drought-resistant crops to cope up with threats of climate changes on livelihoods. Against general perception, the current study reported that due to climate change actually the yield of crops had improved. A similar study was conducted by Moretti et al. (2010) using a positivist ontology to report that farmers experienced a rise in productivity due to climate-related associated awareness which goes in agreement with findings of this study in district Swat and Malakand of Khyber Pakhtunkhwa province in Pakistan. In addition to these, it has been learnt that farmers in this part of the world use indigenous farming knowledge (spoken) which is unwritten to cope up with climate changes in order to adjust their farming practices to sustain their livelihoods. No study is complete in all aspects and that is why we would like to mention the limitations of the study. The biggest limitation pertains to the inclusion of female participants across both phases of data collection which was not possible due to a variety of reasons including cultural barriers of honor and purdah. Future studies can use the findings of the study with a larger sample to further validate the findings of the study in order to mitigate the socioeconomic impact of climate changes tenant farmers in district Swat and Malakand.

## Conclusion

Assessing the socioeconomic risks of climate change on tenant farmers in Pakistan with a longitudinal research design offers important insights regarding coping mechanism of tenant farmers in a flood calamity prone study areas. Detailed information from this research indicates that a tenant farmer was collected to implement adaptive techniques in order to cope with the impact of climate change at farm levels. Findings of the current research study have yielded valuable insights on the level of awareness and strategies used by the farmers for mitigating the impact of the climate change. A key finding pertains to the fact that actually farmers yield had progressively gone up amidst the grand climate risks as indigenous farmers had become more alert to new changes than ever. However, the overall average growth is much less than when compared to global standards. The study highlights that farmers in both districts are affected by climate change due to sudden onset of recurrent natural calamities in the form of cyclones, floods, droughts, hailstorms, and pesticide resistance but overall survival skills and coping strategies had been improved with passage of time. The overall economic losses in study areas are mostly attributed to use of outdated farming practices and lack of awareness regarding climate change due to belief that anthropogenic activities contribute little to climate changes. The study concludes that climate change triggers multiple threats to vulnerable farmers to climate change in socioeconomic terms as they struggle to cope with climate changes, yet they also utilize countermeasures, such as crop rotation, fertilizer change, and sowing date changes to offset the climatic changes. Finally, the study contributes to raise voice for land strapped tenant farmers using a transformative worldview, as these people had been involved in tenant farming for generations despite no or little support from the government. The study recommends introduction of better land distribution legislation and schemes, such as crop sharing and crop insurance to mitigate the effects of climate changes on economically marginalized farmers in district Swat and Malakand.

The study recommends provision of climate resilience training for tenant farmers who had time and again experienced climate volatilities be it nature or otherwise. Then there is also a need for stable agricultural commodity prices monitoring in the market. Other than this, farmers needs to be incentivized and encouraged to improve their knowledge of climate changes by way of training sessions for farmers. Finally, better land distribution legislation and safety schemes, such as crop loan insurance (takaful) in Khyber Pakhtunkhwa, Pakistan.

## Data Availability Statement

The raw data supporting the conclusions of this article will be made available by the authors, without undue reservation.

## Ethics Statement

The studies involving human participants were reviewed and approved by Higher Education Commission, Islamabad, Pakistan (No:21–1,509/SRGP/R&D/HEC/2017). Written informed consent for participation was not required for this study in accordance with the national legislation and the institutional requirements.

## Author Contributions

MY, TS, SK, SU, MN, HH, AZ-M, HM-S, and AV-M contributed to conceptualization, formal analysis, investigation, methodology, and writing and editing of the original draft. All authors contributed to the article and approved the submitted version.

## Conflict of Interest

The authors declare that the research was conducted in the absence of any commercial or financial relationships that could be construed as a potential conflict of interest.

## Publisher’s Note

All claims expressed in this article are solely those of the authors and do not necessarily represent those of their affiliated organizations, or those of the publisher, the editors and the reviewers. Any product that may be evaluated in this article, or claim that may be made by its manufacturer, is not guaranteed or endorsed by the publisher.
